# Clopidogrel Therapy Discontinuation Following Drug Eluting Stent Implantation in Real World Practice in Israel

**DOI:** 10.4021/cr146w

**Published:** 2012-03-20

**Authors:** Miry Blich, Tawfiq Zeidan Shwiri, Sirouch Petcherski, Azriel B Osherov, Haim Hammerman

**Affiliations:** a Department of Cardiology, “Rambam” Health Care Campus and the Technion Medical School, Haifa, Israel

**Keywords:** Clopidogrel Therapy, Discontinuation, Drug Eluting Stent, Implantation

## Abstract

**Background:**

Incidence and predictors of clopidogrel discontinuation after drug eluting stent (DES) implantation, in real world practice, are poorly known.

**Methods:**

Prospective study included all patients who underwent implantation of at least one DES between February 2006 and January 2007. Predictors of clopidogrel discontinuation were assessed by a multivariable analysis.

**Results:**

In 269 patients, mean period for clopidogrel therapy was 13.2 ± 7.2 month. Twenty eight patients (10.4%) discontinued clolopidogrel prematurely (< 3 months). Early clopidogrel discontinuation was a predictor of late stent thrombosis (P = 0.005) and urgent target vessel revascularization (P = 0.05). There was a trend for higher cardiac mortality among that group (P = 0.07). By 12 months, 173 patients (64.3%) have discontinued clopidogrel therapy. The most frequent circumstance to stop clopidogrel before 12 months was recommendation of family physician. Patients that were followed by cardiologist were more encouraged to longer clopidogrel therapy. In multivariable analysis being non Jew (OR 19.2, 95% CI 2.4 to 142, P = 0.005), not followed by cardiologist (OR 4.7, 95% CI 1 to 23.1, P = 0.05) and lack of information regarding the importance of clopidogrel maintenance at discharge from hospital (OR 10.8, 95% CI 2.7 to 42.9, P = 0.001) were independent predictors of early clopidogrel discontinuation.

**Conclusions:**

Clopidogrel discontinuation, in real world practice is not unusual and related to poor outcome. Education for general physicians, clear instructions about the importance of antiplatelet maintenance at discharge and follow up by an expert cardiologist are opportunities to improve adherence do antiplatelet therapy following DES implantation.

## Introduction

Compared with bare metal stents (BMS), drug eluting stents (DES) are associated with significantly less target vessel restenosis and revascularization, though they are associated with higher incidence of very late stent thrombosis (ST) [1, 2]. Although late ST after DES implantation is uncommon, the unpredictability of late ST and its catastrophic clinical consequences make it a concern and have stimulated research to determine the best preventative practices [3, 4]. Dual antiplatelet therapy (DAT) using clopidogrel and aspirin is the standard of care for preventing ST after BMS implantation [5]. Current guidelines recommend that DAT with aspirin and clopidogrel be continued for ≥ 12 months after DES implantation if patients are not at high risk of bleeding [6]. In clinical practice, discontinuation of DAT (especially clopidogrel) after DES implantation is not uncommon and can result in serious cardiovascular events. Tree studies have shown that medical, psychosocial and care process variables may determine the risk for antiplatelet therapy discontinuation [7-9]. Adequate assessment of that complex issue in real life setting all over the world and in Israel is lacking. The objectives of our work were to evaluate the incidence, patients' characteristics and to understand the circumstances associated with premature discontinuation of thienopyridine after implantation of DES in real world practice, in Israel.

## Methods

### Patients

This prospective study included all unselected patients who underwent implantation of at least one DES in Rambam Health Care Campus between February 2006 and January 2007. There were no exclusion criteria.

### Follow up

Patients were interviewed by phone by trained researchers at least 12 months after DES implantation. Patients were asked whether they had been instructed by a hospital doctor to take clopidogrelare at discharge. During the interview, data regarding the follow up was obtained: referral to cardiologist, reasons for stopping or continuing clopidogrel, bleeding events, admissions, surgeries and other invasive procedures. When patients could not be contacted, major efforts were made to contact family members and treating physician. Medical records regarding hospitalization in our hospital or any other hospital in Israel were reviewed. Stent thrombosis (STH) was defined following the recommendation of the Academic Research Consortium ARC [10]. Definite STH required the presence of an acute coronary syndrome with angiographic or autopsy evidence of coronary thrombus or occlusion. Probable STH included patients who experienced unexpected cardiac sudden death or had a ST segment elevation myocardial infarction (STEMI) involving the target –vessel territory. For the purpose of this study total STH was defined as the total of definite and probable STH. Stent thrombosis was then classified as early (0 - 30 days after stent implantation), late (> 31 days).

### Percutaneous coronary intervention (PCI)

This was performed according to standard techniques. IIb/IIIa platelet inhibitors were given according to physician preference. The decision to implant a DES as opposed to a BMS was made according to the instruction of the Israeli Ministry of Health and the recommendation of the Israeli Society of Interventional Cardiology at the time. In general DES as opposed to BMS was implanted for the following indications: 1) lesion at high clinical risk (such as left main lesion); 2) patients at relatively high risk for restenosis (such as patients with diabetes mellitus). The decision to use a specific DES was made according to the operator preference or availability.

### Antiplatelet prescription

Patients not receiving antiplatelet treatment prior to DES implantation were given aspirin ≥ 100 mg prior to the procedure and clopidogrel ≥ 300 mg prior to or immediately after the procedure. All patients were advised to maintain aspirin 100 mg qd lifelong and to take clopidogrel 75 mg qd for one year.

### Statistical analysis

Quantitative variables are presented as mean ± standard deviation. Two group comparisons were performed using the Fisher exact test or Person Chi square for categorical variables and Mann-Whitney U test for quantitative variables. All p values were two tailed with statistical significance set at 0.05. Predictors with a P value ≤ 0.1 were entered in a multivariate logistic regression analysis, to test independence of these predictors. Analysis was performed using SPSS software version 15.0.

## Results

### Baseline characteristics

The study population included 314 consecutive patients receiving 436 DES. Clinical characteristics are listed in Table 1. Sirolimus Eluting stents (SES) were implanted in 194 lesions in 149 patients. Paclitaxel Eluting Stents (PES) was implanted in 181 lesions in 133 patients and Zotarolimus Eluting Stents (ZES) in 61 lesions in 50 patients. All stents were deployed successfully. There were no significant differences between the stent groups regarding procedural complications and in hospital outcome. Twenty seven patients (8.5%) received one or more BMS in addition to the DES.

**Table 1:** Baseline Characteristics of the Study Population (n = 314)

Variable	Number/Mean ± SD	Percentage
Male sex	260	82.8
Age, years	60 ± 10.5	
Jew	216	68.8
High school education	67	21.3
Receives National Insurance Benefits	32	10.2
Hypertension	205	65.3
Diabetes Mellitus	138	43.9
Current smoker	102	32.5
Body mass index	29.5 ± 5.5	
LDL level mg/dL	109.3 ± 35.8	
Previous myocardial infarction	109	34.7
Previous thromboembolic event	17	5
Acute coronary syndrome,	188	59.8
STEMI	40	12.7
LVEF < 40%	37	11.7
Multivessel disease	194	61.8
Refferal to Cardiologist	163	52
Instruction by doctor at discharge	224	71

Data are expressed as mean ± SD or as number (percentage). LDL: low density lipoprotein; STEMI: ST elevation myocardial infarction; LVEF: left ventricular ejection fraction on echocardiography.

### Mode of follow up

Two hundred thirty eight patients (76%) were interviewed by phone. Thirty nine patients (12%) were followed by interviewing their family physician and the rest thirty seven patients (12%) were followed by reviewing their medical records.

### Antiplatelet therapy at discharge and during the follow up

**Figure 1:**
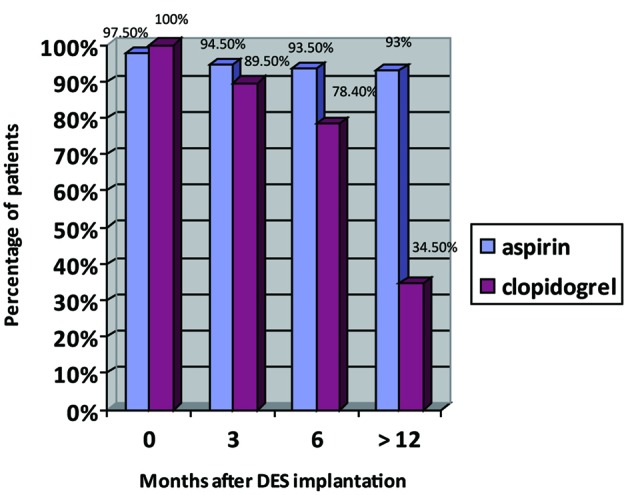
Antiplatelet therapy during the follow up, n = 269.

**Figure 2:**
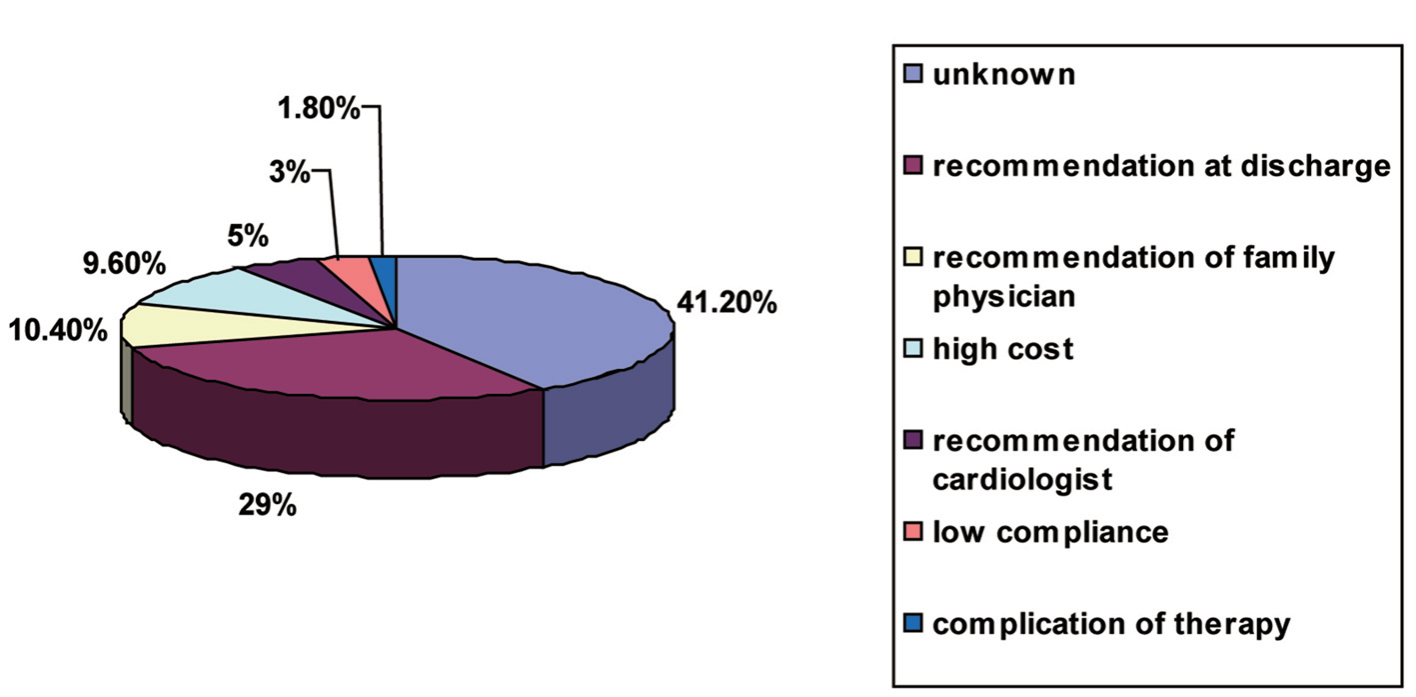
Distribution of reasons for stopping clopidogret therapy during the follow up, n = 269.

At discharge all patients were treated with clopidogrel and 97.5% were on DAT (eight patients were not treated with aspirin due to gastrointestinal problems). Information regarding the exact time for clopidogrel discontinuation during the follow up was available for 269 patients. Mean follow up was 20 ± 6.7 months. Mean period for clopidogrel therapy was 13.2 ± 7.2 month (median 12 month). Prevalence of DAT during the study period is presented in Figure 1. Twenty eight patients (10.4%) discontinued clopidogrel therapy within 3 months of stent implantation. By 12 months, 173 patients (64.3%) have discontinued clopidogrel therapy. The circumstance associated with clopidogrel discontinuation could be assessed in 134 patients (Fig. 2). The most frequent circumstance was adherence to discharge recommendation (which was 12 months of therapy) (n = 67). The most frequent circumstance to stop clopidogrel before 12 month was recommendation of family physician (n = 24) and the high cost of the drug (n = 22). Among twenty eight patients that discontinued clopidogrel before 3 month, the most frequent and known circumstance was recommendation of family physician (n = 7), high cost of the drug (n = 3) and low compliance (n = 3). Among patients that continued clopidogrel therapy more than 12 months, the most frequent circumstances were recommendation by the cardiologist during the follow up (42 patients), contra indication to aspirin (11 patients) and recommendation by the family physician (8 patients).

### Outcome during the follow up and early clopidogrel discontinuation

Among twenty eight patients that discontinued clopidogrel prematurely (< 3 months), four patients had definite or probable stent thrombosis (14%), two patients had urgent target vessel revascularization (7%) and two patients died (7%). Clopidogrel therapy of less than 3 months was a predictor of late stent thrombosis (P = 0.005) and urgent target vessel revascularization (P = 0.05). There was a trend for higher cardiac mortality among that group (P = 0.07).

### Predictors of early antiplatelet discontinuation

Predictors of early (< 3 month) clopidogrel therapy discontinuation are presented in Table 2. Being non high educated and unemployment predicted a higher risk of early clopidogrel discontinuation according to univariable analysis. In multivariable analysis being non Jew, not followed by cardiologist and lack of information regarding the importance of clopidogrel maintenance at discharge from hospital were independent predictors of early clopidogrel discontinuation (Table 2). Low compliance and high cost of the drug were not found risk factors for clopidogrel discontinuation.

**Table 2:** Multiple Logistic Regression Analysis for Predictors of Early Discontinuing Clopidogrel Therapy Within 3 Months

Variable	Univariable analysis		Multivariable analysis	
	OR (95% CI)	P	OR (95% CI)	P
Non Jew	13.6 (3.6 - 51.1)	0.001	19.2 (2.4 - 142)	0.005
No high education	3.7 (0.8 - 16.1)	0.05	2.2 (0.2 - 24.4)	0.5
National insurance benefits (unemployed)		0.003	1.3 (0.1 - 13.4)	0.8
Not referred to cardiologist	2.2 (1 - 5.3)	0.087	4.7 (1 - 23.1)	0.05
No medication instruction by doctor at discharge	7.5 (3.3 - 17.1)	< 0.0001	10.8 (2.7 - 42.9)	0.001

OR: odds ratio; CI: confidence interval.

### Antiplatelet therapy and bleeding during the follow up

Sixty two patients (19.7%) on DAT therapy experienced bleeding events. Eighty five percent of those events were minor bleeding. Seven patients (2.2%) were hospitalized and 6 patients (1.9%) required blood transfusions. Five patients (1.6%) stopped clopidogrel therapy due to bleeding. During the follow up period 26 patients (8.3%) underwent invasive procedures. Twelve patients (46%) stopped clopidogrel therapy before the procedure. One of the fourteen patients that did not stop clopidogrel before endoscopic colon polypectomy was admitted with acute rectal bleeding and blood transfusions were required.

## Discussion

In real life setting Israeli patients, median period for clopidogrel therapy after DES implantation was 12 months. This was most common due to adherence to discharge recommendation from hospital. Ten percent of patients receiving DES interrupted clopidogrel therapy within 3 months after implantation most commonly due to medical recommendation of family physician. High cost of the drug and low compliance were two other less common causes for early clopidogrel discontinuation (< 3 month). As reported by others [11], we found that early cessation of clopidogrel may be related to poor outcome. Twenty two percent of patients and 64% percent of patients stopped clopidogrel within 6 and 12 months of implantation, respectively. The most frequent circumstance to stop clopidogrel before 12 months was recommendation of family physician. Patients that were followed by cardiologist were more encouraged to longer clopidogrel therapy. The incidence of clopidogrel discontinuation in our population is similar to results from article by Ho, which found that 20% of patients had discontinued clopidogrel therapy within 6 months of receiving stent [12]. Results from the Prospective Registry Evaluating Myocardial infarction: Events and Recovery Registry showed that 14% of acute coronary syndrome patients had discontinued DAT within the first 30 days following placement of DES [7]. Among 3401 patients that underwent stent implantation in Hadassah Medical Center, 60% stopped clopidogrel prematurely (less than 1 month following bare metal stenting and less than 6 months treatment following use of a drug eluting stent) [13].

Few studies have addressed the rate of DAT after DES implantation and its determinants [7-9]. Spertuz and Poh Showed that social factors such as not completing high school [7] or living alone [8] may be associated with antiplatelet discontinuation. In our work low education and unemployment were predictors in univariate analysis. In the study by Ferreira Gonzalez as in the present study, lack of discharge instructions was found an independent predictor of antiplatelet therapy discontinuation [7, 9]. According to our and the study by Ferreira Gonzalez [9] clopidogrel discontinuation could not be justified by the occurrence of major hemorrhage or surgical procedure. Being non Jew was found an independent factor for early discontinuation of clopidogrel. This may be related to lower socio economic status.

The incidence rate of antiplatelet discontinuation was higher from month 6 - 12 than from discharge to month 6 (Fig. 1). Patients that were not followed by cardiologist were at risk to stop clopidogrel earlier. This may illustrate the impression among family physicians of lesser risk associated with clopidogrel interruption beyond the sixth month after DES implantation.

### Conclusions

Clopidogrel discontinuation, in real world practice, in Israeli population is not unusual during the first year after DES implantation. Clopidogrel discontinuation was rarely associated with major bleeding, but more often associated with medical advice, especially at the discharge from hospital and of the family physician. Lack of discharge instructions about the importance of maintaining antiplatelet therapy and absence of a follow up by cardiologist are independent predictors of early clopidogrel discontinuation. Opportunities to improve adherence do antiplatelet therapy following implantation of DES include education for general physicians and patients, clear instructions about the importance of antiplatelet maintenance at discharge and follow up by an expert cardiologist.
